# The actin-binding protein palladin associates with the respiratory syncytial virus matrix protein

**DOI:** 10.1128/jvi.01435-24

**Published:** 2024-10-03

**Authors:** Shadi Shahriari, Reena Ghildyal

**Affiliations:** 1 Biomedical Research Cluster, Faculty of Science and Technology, University of Canberra, Canberra, Australia; Loyola University Chicago - Health Sciences Campus, Maywood, Illinois, USA

**Keywords:** protein-protein interactions, microfilaments, cytoskeleton, viral budding and release, virus-host interactions, viral components, respiratory syncytial virus, viral structures

## Abstract

The respiratory syncytial virus (RSV) matrix (M) protein plays an important role in infection as it can interact with viral components as well as the host cell actin microfilaments. The M–actin interaction may play a role in facilitating the transportation of virion components to the apical surface, where RSV is released. We show that M protein’s association with actin is facilitated by palladin, an actin-binding protein. Cells were infected with RSV or transfected to express full-length M as a green fluorescent protein (GFP)-tagged protein, followed by removal of nuclear and cytosolic proteins to enrich for cytoskeleton and its associated proteins. M protein was present in inclusion bodies tethered to microfilaments in infected cells. In transfected cells, GFP-M was presented close to microfilaments, without association, suggesting the possible involvement of an additional protein in this interaction. As palladin can bind to proteins that also bind actin, we investigated its interaction with M. Cells were co-transfected to express GFP-M and palladin as an mCherry fluorescent-tagged protein, followed by cytoskeleton enrichment. M and palladin were observed to colocalize towards microfilaments, suggesting that palladin is involved in the M–actin interaction. In co-immunoprecipitation studies, M was found to associate with two isoforms of palladin, of 140 and 37 kDa. Interestingly, siRNA downregulation of palladin resulted in reduced titer of released RSV, while cell associated RSV titer increased, suggesting a role for palladin in virus release. Together, our data show that the M–actin interaction mediated by palladin is important for RSV budding and release.

**IMPORTANCE:**

Respiratory syncytial virus is responsible for severe lower respiratory tract infections in young children under 5 years old, the elderly, and the immunosuppressed. The interaction of the respiratory syncytial virus matrix protein with the host actin cytoskeleton is important in infection but has not been investigated in depth. In this study, we show that the respiratory syncytial virus matrix protein associates with actin microfilaments and the actin-binding protein palladin, suggesting a role for palladin in respiratory syncytial virus release. This study provides new insight into the role of the actin cytoskeleton in respiratory syncytial virus infection, a key host–RSV interaction in assembly. Understanding the mechanism by which the RSV M protein and actin interact will ultimately provide a basis for the development of therapeutics targeted at RSV infections.

## INTRODUCTION

The RSV matrix protein (M) and the actin cytoskeleton (microfilaments) play important roles in the assembly and budding of RSV (1, 2). The M protein plays a role in viral assembly through its interactions with other RSV structural proteins and serves as a bridge between the ribonucleoprotein (RNP) and the envelope protein complexes (3). Previously, we have shown that the microfilament network is required in RSV infection for optimal assembly and that RSV M interacts with the actin microfilament network (4). M and actin may play a role in facilitating the transportation of the RNPs to assembly sites (3), and indeed, M protein is able to bind directly to polymerized actin *in vitro*. In addition, although a complex containing the N, P, and M2-1 protein can bind polymerized actin, M is required to anchor the complex onto the microfilament network (2, 5).

In this study, we have further characterized the interaction of M protein with actin microfilaments. Interestingly, enrichment for the cytoskeleton in RSV-infected cells resulted in the localization of M protein toward the microfilaments without direct association. Many actin-binding proteins (ABPs) have the ability to bind proteins that bind actin (6) and can act as scaffolding proteins to recruit such proteins to the microfilament network (7). Consequently, we hypothesized that an ABP may be mediating the association of M with microfilaments.

The Akt serine/threonine kinase family includes three homologous isoforms that have a widely diverse repertoire of downstream effects in different settings by targeting a large number of substrates (8). Akt is ubiquitous in the cytosol and nucleus and is activated at the plasma membrane (9, 10). Akt regulates various cellular processes, namely cell survival, apoptosis, proliferation, mobility, and the PI3K pathway (11, 12). Akt is also involved in actin cytoskeleton organization through one of its substrates, palladin (13–15).

The Akt substrate palladin has a role in the regulation and organization of the actin cytoskeleton (14, 16, 17). Palladin has many isoforms; however, only three have been characterized, 90, 140, and 200 kDa (18); the 90 kDa isoform of palladin is an F-actin-binding protein (19). The other isoforms have been suggested to be established through alternate splicing in specific cell types (18). Palladin expression levels are shown to be lower in adult tissues, supporting the notion that palladin isoforms may be involved in the cytoskeletal organization of differentiating cells (19). Palladin, an actin-crosslinking protein itself, colocalizes with actin-rich structures in several cell types and functions as a molecular scaffold for various ABPs (14, 16, 17). This way, palladin is able to undertake its scaffolding role for the organization of the cytoskeleton (19).

In this study, we show that palladin associates directly with M protein in RSV-infected cells and in cells co-transfected to express mCherry–palladin-C-7 and GFP-M. Interestingly, M pulls down an ~37 kDa isoform of palladin, while displacing an ~140 kDa isoform. Clearly, the M–palladin interaction is complex and involves at least two palladin isoforms. Although over-expression of mCherry–palladin or silencing of palladin did not have any effect on the localization of M protein, silencing resulted in reduced titer of virus released into the culture supernatant.

## RESULTS

### M associates with microfilaments indirectly in cell culture

**Fig 1:**
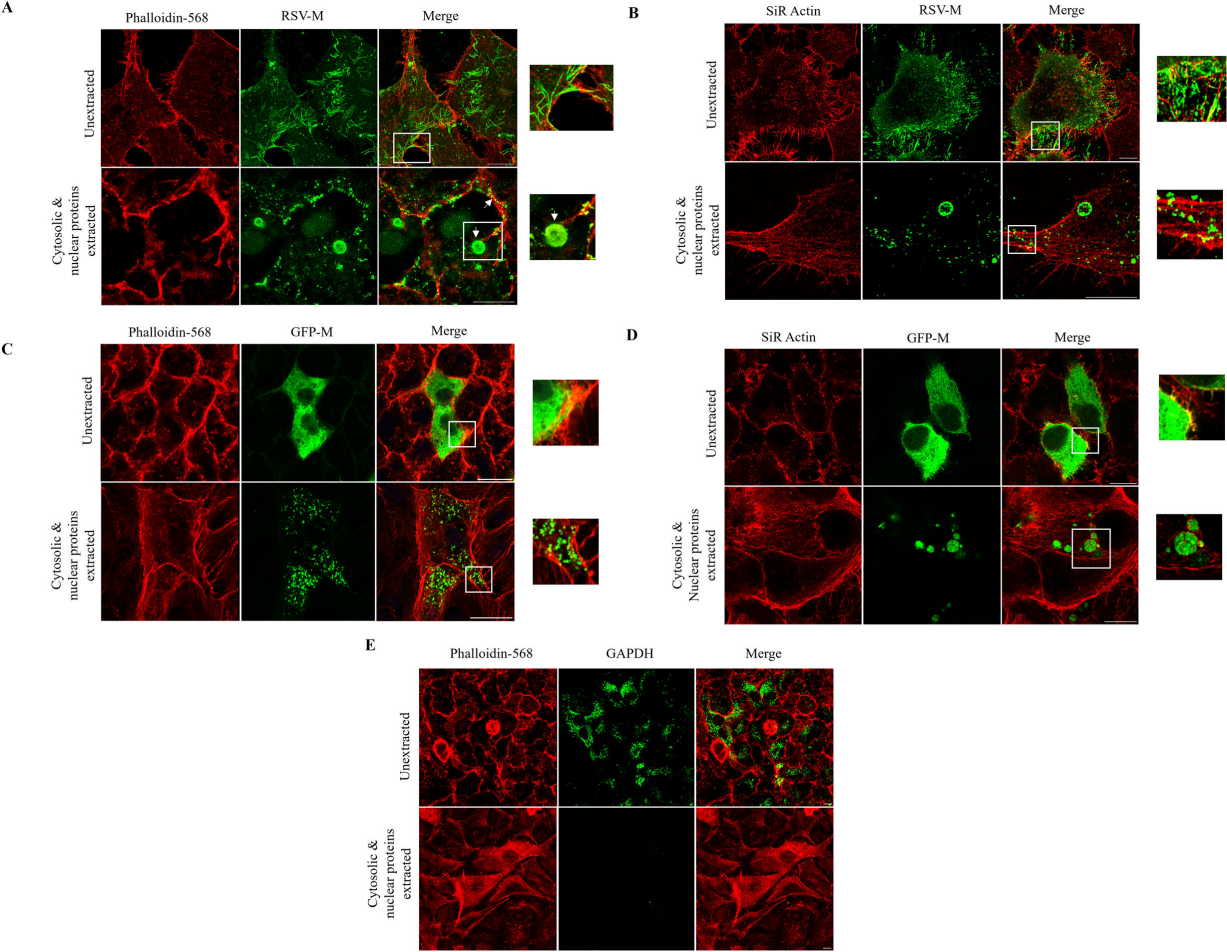
M associates with the microfilament network indirectly in infected and transfected cells. (**A**) Vero cells were cultured overnight before infection with RSV A2 at MOI of 1. Cytoskeleton enrichment was undertaken 24 h p.i. for the removal of cytosolic and nuclear proteins (images labeled cytosolic and nuclear proteins extracted). Cells were probed for the visualization of the microfilament network (phalloidin-568; red) and RSV M (green). Images were acquired by super-resolution-stimulated emission depletion microscopy (STED) microscopy. Computer-generated merged images of the two channels are also shown (images labeled merge). Inclusion bodies are indicated by white arrows. Zoomed in images of indicated sections are shown on the right. Scale bars = 10 µM. (**B**) A549 cells were cultured, infected, and treated for cytoskeleton enrichment as in (**A**). Cells were probed for the visualization of the microfilament network (SiR Actin; red) and RSV M (green). The colocalization of M and actin microfilaments is indicated in yellow (image labeled merge). Images were acquired by STED microscopy. Zoomed in images of indicated sections are shown on the right. Scale bars = 10 µM. (**C**) Vero cells were cultured overnight and transfected to express GFP-M (green). Cytoskeleton enrichment was undertaken 24 h p.i. as in (**A**) and cells probed for the visualization of the microfilament network (phalloidin-568; red). Images were acquired by confocal microscopy. Computer-generated merged images of the two channels are also shown (images labeled merge). Zoomed in images of indicated sections are shown on the right. Scale bars = 10 µM. (**D**) A549 cells were cultured, transfected, and treated for cytoskeleton enrichment as in (**C**) and probed for the visualization of the microfilament network (SiR Actin; red). Images were acquired by STED microscopy. Computer-generated merged images of the two channels are also shown (images labeled merge). Zoomed in images of indicated sections are shown on the right. Scale bars = 10 µM. (**E**) A549 cells were cultured overnight and mock transfected. Cytoskeleton enrichment was undertaken 24 h later for the removal of cytosolic proteins as in (**C**). Cells were probed for the visualization of the cytosolic proteins (GAPDH; green) and the microfilament network (phalloidin; red). Images were acquired by confocal microscopy. Scale bar = 10 µM. All data shown are representative of two independent experiments.

Vero cells were infected with RSV A2 at multiplicity of infection (MOI) of 1 followed by cytoskeleton enrichment by extracting nuclear and cytosolic proteins 24 h post-infection (p.i.) (Fig. 1A). Infected cells were stained for the visualization of microfilaments (images labeled phalloidin-568, shown in red) and the M protein (images labeled RSV M, shown in green), and visualized by super-resolution-stimulated emission depletion microscopy (STED). RSV M colocalized with thick viral microfilaments at the cell periphery (labeled unextracted, shown in yellow in images labeled merge, and detail shown in zoomed image). Following cytoskeleton enrichment, M was localized at the large inclusion bodies (IBs; shown in images labeled cytosolic & nuclear proteins extracted at white arrows) and appeared to be tethered to thin microfilaments (labeled phalloidin-568, shown in red) as opposed to the thicker microfilaments at the periphery. M was also localized at small puncta, possibly small IBs, that were present along the thicker peripheral microfilaments (white arrow). Some limited colocalization of M with microfilaments was observed at these puncta, denoted by the occasional yellow color in the merge image. Similar results were obtained in RSV-infected A549 cells (Fig. 1B). SiR Actin was used for the visualization of microfilaments (shown in red) in A549 cells.

Colocalization between actin (phalloidin or SiR Actin signal) and RSV M was confirmed by quantitative analysis to determine Pearson’s correlation coefficient (PCC; r) and Mander’s coefficients (MCs; M1 and M2) using ImageJ and are shown in Table 1. The PCC is used to determine the overlap between two images by measuring their pixel-by-pixel signal covariance, normalized by the product of their standard deviations, assuming a linear relationship. PCC values range from −1 to 1, with a value of −1 demonstrating that the fluorescence intensities of the two images are perfectly linearly unrelated, while a value of 1 demonstrates that the fluorescence intensities are perfectly linearly related; a value of 0 indicates that there is no correlation (20, 21). Therefore, the PCC assumes that the two proteins are functionally related and that their quantities will also be related. The Manders’ coefficients (MCs; M1 and M2) are used to quantify the co-occurrence of two molecules of interest in a particular location within a cell and are used to calculate the percentage of the intensity from one channel (first image) that overlaps the intensity from the other channel (second image) (22). Therefore, the MCs determine the degree of spatial overlap between the two proteins (21). Quantitative analysis identified a weak positive correlation (r = 0–0.3) between actin (phalloidin signal) and RSV M in both unextracted and extracted infected Vero cells (r = 0.21 and 0.22, respectively). The degree of overlap of the actin and RSV M signals was determined to be 0.34 (M1; actin) and 0.66 (M2; RSV M) in unextracted Vero cells, showing that ~66% of green pixels (RSV M) co-occurred with the red pixels (actin/phalloidin), and ~34% of red pixels co-occurred with green pixels. In extracted cells, the degree of overlap was determined to be 0.72 and 0.28, demonstrating that ~72% of green pixels co-occurred with the red pixels and ~28% of red pixels co-occurred with green pixels.

**TABLE 1:** Colocalization analysis between actin and RSV M in infected Vero and A549 cells

Cell line	Treatment	Pearson’s correlation coefficient (r)	Mander’s coefficient 1 (actin; red signal)	Mander’s coefficient 2 (RSV-M; green signal)
Vero	Unextracted	0.21	0.34 (34%)	0.66 (66%)
	Extracted	0.22	0.28 (28%)	0.72 (72%)
A549	Unextracted	0.21	0.43 (43%)	0.57 (57%)
	Extracted	0.15	0.38 (38%)	0.62 (62%)

In A549 cells, a weak positive correlation (0–0.3) between actin (SiR Actin signal) and RSV M was also identified in both unextracted and extracted infected cells (r = 0.21 and 0.15, respectively). Mander’s coefficients of 0.43 (M1; actin) and 0.57 (M2; RSV M) showed that ~43% of red pixels co-occurred with green pixels, while 57% of green pixels co-occurred with the red pixels in unextracted cells. In extracted A549 cells, ~38% (0.38, M1; actin) of red pixels overlapped with the green pixels, while ~62% (0.62, M2; RSV M) of green pixels overlapped with red pixels. Taken together, these results demonstrate that RSV M associates with actin microfilaments in RSV infected Vero and A549 cells.

We next investigated whether RSV M colocalizes with microfilaments when expressed by itself in transfected cells. GFP-M was expressed in Vero or A549 cells, followed by cytoskeleton enrichment 24 h post-transfection (p.t.) and confocal laser scanning microscopy (CLSM). GFP-M was dispersed in the cytoplasm in untreated and unextracted Vero cells (Fig. 1C, labeled unextracted, in green). Similar to the localization of RSV M in infected cells (Fig. 1A and B), GFP-M colocalized with microfilaments at the cell periphery (Fig. 1C, indicated by the color yellow in the image labeled unextracted merge). As the other IB constituent proteins are not present, no inclusions were observed. Subsequent to cytoskeletal enrichment, GFP-M was observed to form punctate structures towards the cell periphery and microfilaments, without direct association with the microfilaments (Fig. 1C, labeled cytosolic and nuclear proteins extracted, in green). Similar results were obtained in GFP-M-transfected A549 cells (Fig. 1D). GFP-M was presently dispersed throughout the cytoplasm, with colocalization with microfilaments at the cell periphery in unextracted cells (Fig. 1D, labeled unextracted, colocalization shown in yellow in image labeled merge). Upon cytoskeletal enrichment, M formed puncta, which appeared to localize close to thin microfilaments (Fig. 1D, labeled cytosolic and nuclear proteins extracted, merge), similar to the localization of RSV M in infected cells, suggesting an indirect association.

Pearson’s (r) and Mander’s coefficients (M1 and M2) were calculated as above and are shown in Table 2. A weak positive correlation (0–0.3) between actin (phalloidin signal) and GFP-M in both unextracted and extracted Vero cells (r = 0.22 and 0.19, respectively) was determined. Mander’s coefficients of 0.30 (M1; actin) and 0.70 (M2; GFP-M) in unextracted Vero cells demonstrated that ~70% of green pixels (GFP-M) colocalized with the red pixels (actin/phalloidin), and ~30% of red pixels colocalized with green pixels. In extracted cells, Mander’s coefficients of 0.94 and 0.06 showed that ~94% of green pixels co-occurred with the red pixels, and ~6% of red pixels co-occurred with green pixels. A weak positive correlation (0–0.3) between actin (SiR Actin signal) and GFP-M was also demonstrated in unextracted (r = 0.08) and extracted (r = 0.01) A549 cells, respectively. Mander’s coefficients of 0.77 (M1; actin) and 0.23 (M2; GFP-M) in unextracted cells and 0.08 (M1; actin) and 0.92 (M2; GFP-M) in extracted cells showed that ~77% of red pixels colocalized with green pixels, and ~23% of green pixels colocalized with red pixels in unextracted cells, while ~8% of the red pixels co-occurred with green pixels, with majority of the green pixels (~92%) co-occurring with the red pixels in extracted cells. These results demonstrate that not all actin is associating with M as expected, instead M is interacting with actin.

**TABLE 2:** Colocalization analysis between actin and M in Vero and A549 cells transfected with GFP-M

Cell line	Treatment	Pearson’s correlation coefficient (r)	Mander’s coefficient 1 (actin; red signal)	Mander’s coefficient 2 (GFP-M; green signal)
Vero	Unextracted	0.22	0.30 (30%)	0.70 (70%)
	Extracted	0.19	0.06 (6%)	0.94 (94%)
A549	Unextracted	0.08	0.77 (77%)	0.23 (23%)
	Extracted	0.01	0.08 (8%)	0.92 (92%)

The process of cytoskeletal enrichment results in the removal of all proteins that are not attached to the cytoskeleton. That M was detectable in the enriched samples (both in infected and in transfected cells) confirms that it is associating with the cytoskeleton. As RSV M was seen to localize towards the microfilaments without direct association with the network, our data suggest an indirect association between RSV M and actin microfilaments in cell culture. This further suggests that the RSV M–microfilament interaction may be facilitated by an ABP that can also bind to proteins that associate with actin, including RSV M.

Regardless of differences due to the cell line used, a significant proportion of the total cellular GFP-M colocalized with microfilaments. It is important to note that Vero cells were used initially for proof of concept as RSV has been shown to induce effective infection in this cell line (23). However, as A549 cells are more relevant for this study, they were used to elucidate the M–actin interaction in RSV infection, and for all subsequent experiments.

That extraction of nuclear and cytosolic proteins effectively resulted in the enrichment of the cytoskeleton is shown in Fig. 1E. A549 cells were either left untreated or were enriched for cytoskeleton and associated proteins, followed by staining for the microfilament network (images labeled phalloidin-568, shown in red) and GAPDH (images labeled GAPDH, shown in green). The microfilament network was effectively enriched as shown by the ability to visualize the filaments within the cells and the loss of GAPDH following treatment (compare unextracted and cytosolic and nuclear proteins extracted).

### Palladin colocalizes with RSV M protein and microfilaments

**Fig 2:**
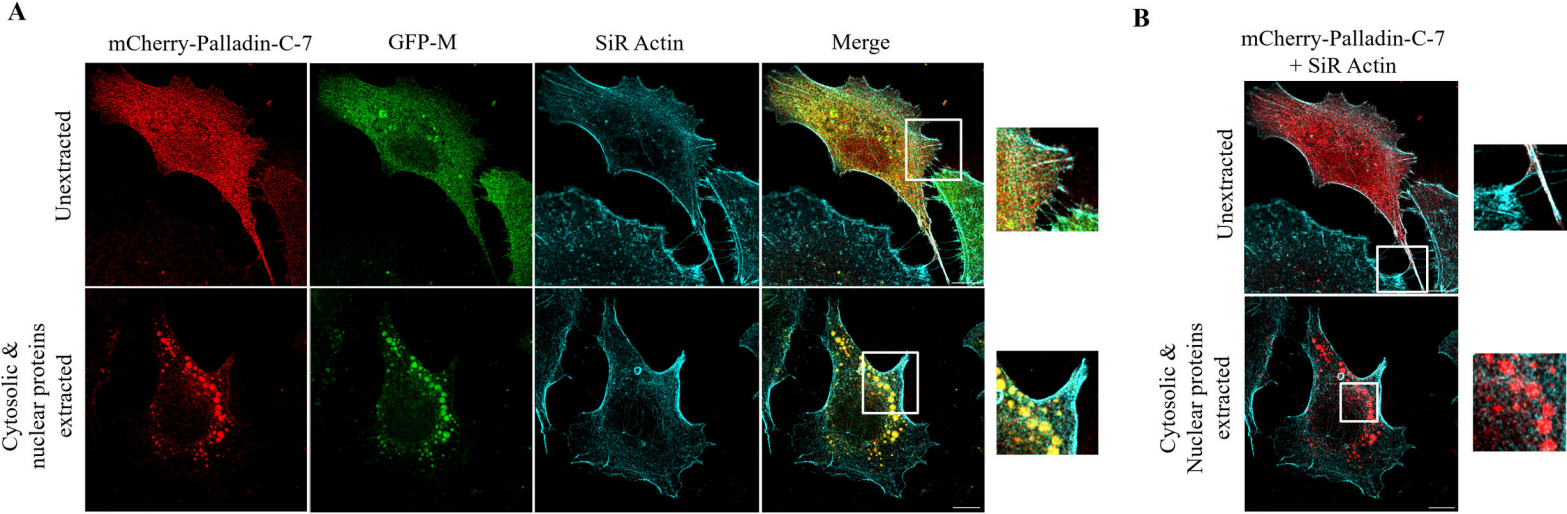
Palladin associates and colocalizes with M and microfilaments in A549 cells. (**A**) Cells were cultured overnight and transfected to express mCherry-Palladin-C-7 and GFP-M. Transfected cells were either left untreated (images labeled unextracted) or treated (images labeled cytosolic and nuclear proteins extracted) with enrichment buffers to remove soluble nuclear and cytosolic proteins. Cells were probed for the visualization of the microfilament network (SiR Actin; in cyan). The colocalization of palladin (in red) and M (in green) is indicated in yellow (image labeled merge). Zoomed-in images of indicated sections are shown on the right. Scale bars = 10 µM. Additional images including zoomed-in images of extracted cells are included as supplemental material (Fig. S1 and S2, respectively). (**B**) Images acquired in (**A**) were overlayed to visualize the colocalization of palladin and actin in cytoskeleton-enriched cells (shown in white, and detail shown in zoomed image). All data shown are representative of two independent experiments.

Palladin is an ABP that has a role in actin remodeling and functions as a scaffolding protein, recruiting other actin-associated proteins to microfilaments (13, 24). Previously, we have shown that M is an ABP capable of direct association with polymerized actin *in vitro* (4). Taken together with the data shown in Fig. 1, this led to our hypothesis that palladin may be facilitating M protein’s association with microfilaments in infected cells. We tested this hypothesis in the transfected cell system. A549 cells were co-transfected to express mCherry-Palladin-C-7 and GFP-M, followed by cytoskeleton enrichment and immunostaining 24 h p.i., and visualization by STED microscopy (Fig. 2A). The mCherry-Palladin-C-7 construct contains ~72 kDa of the C-terminal sequence of palladin, and the mCherry fusion tag is located at the N terminal on the mCherry-C-1 backbone. Mouse palladin has six isoforms, and the mouse palladin sequence included in this construct corresponds to mouse isoform 3. GFP-M was mostly cytoplasmic without treatment (image labeled unextracted GFP-M), as expected based on our data shown in Fig. 1. mCherry-Palladin-C-7 appeared both nuclear and cytoplasmic (image labeled unextracted mCherry-Palladin-C-7), however, with more localization in the cytoplasm. Microfilaments lined the cell periphery and were observed within the cell (images labeled SiR Actin in cyan), remaining unchanged upon extraction. Both GFP-M and mCherry-Palladin-C-7 proteins remained cytoplasmic upon removal of cytosolic and nuclear proteins, and subsequent cytoskeletal enrichment, suggesting that both proteins associate with the microfilament network. Merged images show that GFP-M colocalizes with mCherry-Palladin-C-7 in the cytoplasm in untreated cells (indicated by yellow in the image labeled unextracted, merge) and aggregates towards microfilaments in extracted cells (indicated in yellow in the image labeled cytosolic and nuclear proteins extracted, merge and zoomed image).

To confirm colocalization between M and palladin, Pearson’s correlation coefficient (PCC, r) and Mander’s coefficients (M1 and M2) were calculated using ImageJ and are shown in Table 3. There was a strong positive correlation (0.60–0.79) between GFP-M and mCherry-Palladin-C7 in unextracted (r = 0.71) and extracted cells (r = 0.70), respectively. Mander’s coefficients of 0.53 (M1; mCherry-Palladin-C-7) and 0.47 (M2; GFP-M) determined the degree of co-occurrence in extracted cells, showing that ~47% of green pixels (GFP-M) colocalized with the red pixels (mCherry-Palladin-C-7), and ~53% of red pixels colocalized with green pixels. In extracted cells, Mander’s coefficients of 0.43 and 0.57 suggested a somewhat different degree of co-occurrence as unextracted cells, with more green pixels co-occurring with red pixels in extracted cells; ~57% of green pixels co-occurred with the red pixels, and ~43% of red pixels co-occurred with green pixels. This suggests that the association between palladin and M is almost equal, that is, one protein is not interacting with the other more significantly.

**TABLE 3:** Colocalization analysis between palladin and M in A549 cells

	Pearson’s correlation coefficient (r)	Mander’s coefficient 1 (mCherry-Palladin-C-7; red signal)	Mander’s coefficient 2 (GFP-M; green signal)
Unextracted	0.71	0.53 (53%)	0.47 (47%)
Extracted	0.70	0.43 (43%)	0.57 (57%)

It is important to note that actin microfilaments are shown in cyan to visualize colocalization effectively; as SiR Actin emits light at 674 nm (far red), and mCherry between 600 nm and 680 nm (red), it is difficult to determine colocalization qualitatively. Additionally, colocalization analysis in ImageJ utilizes green and red pixels and, as such, while the SiR Actin channel in Fig. 2 is cyan, it was analyzed as green.

Actin and mCherry-Palladin-C-7 colocalize within the cytoplasm along the cytoskeleton network and at the cell periphery, including in filaments in untreated cells (Fig. 2B, indicated by white in the image labeled unextracted and zoomed image). Upon enrichment, palladin is in the cytoplasm, localized towards the cell periphery as aggregates embedded within the microfilament network (image labeled cytosolic and nuclear proteins extracted and detail shown in zoomed image).

Colocalization between palladin and actin was analyzed as above to determine Pearson’s correlation coefficient (PCC, r) and Mander’s coefficients, and are shown in Table 4. As expected, there was a strong to moderate positive correlation between mCherry-Palladin-C7 and microfilaments (actin) in unextracted cells (r = 0.67) and extracted cells (r = 0.45), respectively. Mander’s coefficients of 0.49 (M1; actin) and 0.51 (M2; mCherry-Palladin-C-7) showed that ~49% of green pixels (actin) colocalized with the red pixels (mCherry-Palladin-C-7), and ~51% of red pixels co-occurred with green pixels in unextracted cells. In extracted cells, Mander’s coefficients of 0.48 and 0.51 suggested approximately the same degree of co-occurrence as unextracted cells, showing that ~48% of green pixels co-occurred with the red pixels and ~51% of red pixels co-occurred with green pixels.

**TABLE 4:** Colocalization analysis between actin and palladin in A549 cells

	Pearson’s correlation coefficient (r)	Mander’s coefficient 1 (mCherry-Palladin-C-7; red signal)	Mander’s coefficient 2 (actin; green signal)
Unextracted	0.49	0.53 (53%)	0.47 (47%)
Extracted	0.40	0.67 (67%)	0.33 (33%)

### Palladin interacts with RSV M

**Fig 3:**
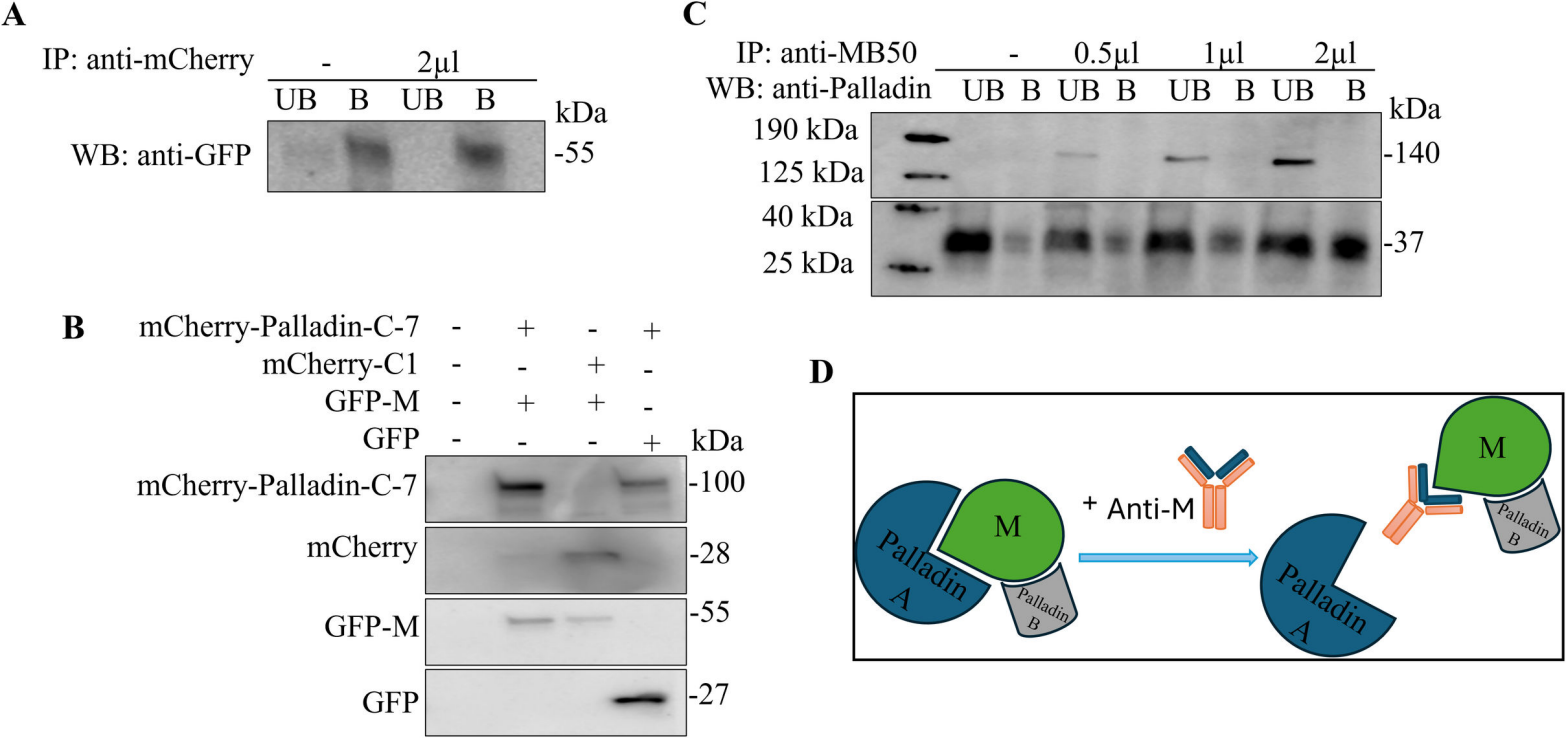
Palladin forms complexes with M in infected and transfected cells. (**A**) Overnight sub-confluent monolayers were transfected to express mCherry-Palladin-C-7 and GFP-M proteins. At 24 h p.t., cells co-expressing mCherry-Palladin-C-7 and GFP-M were collected by FACS and lysed. Samples were used in a co-immunoprecipitation study to determine the palladin–M interaction through complex formation. Primary anti-mCherry antibody was either not added (labeled -) or added (2 µL) to lysates and immune complexes collected with EZview Red Protein G Affinity Gel. Unbound (labeled UB) and bound (labeled B) proteins were analyzed by Western blot analysis and probed for GFP. Full Western blot images are provided as supplemental material (Fig. S3). (**B**) Overnight sub-confluent monolayers were transfected and collected as in (**A**). Samples were analyzed by Western blot and proteins of interest (mCherry, mCherry-Palladin-C-7, GFP-M, and GFP) were detected by probing for GFP and mCherry. (**C**) Overnight sub-confluent monolayers were infected with RSV A2 at a MOI of 1. Lysates were collected 16–18 h p.i. and used in a co-immunoprecipitation as in (**A**). Primary anti-M antibody was either not added (labeled -) or added in increasing amounts (0.5, 1, and 2 µL) to lysates. Unbound (UB) and bound (**B**) fractions were analyzed by Western blot and probed for palladin. All data shown are representative of two independent experiments. (**D**) Model. We hypothesize that a complex between M and two isoforms of palladin is formed; however, with increasing primary anti-M antibody, the higher molecular weight palladin dissociates and the complex with the lower molecular-weight palladin, and M is pulled down and detected.

A co-immunoprecipitation assay was undertaken to investigate the interaction between M and palladin in both transfected and RSV infected cells. In transfected cells, GFP-M was co-precipitated with mCherry–palladin (Fig. 3A). In the absence of anti-mCherry antibody, GFP-M was observed in both the unbound (labeled UB) and bound (labeled B) fractions at 55 kDa, moving completely into the bound fraction when mCherry–palladin was immunoprecipitated. Western blot analysis confirmed the expression of mCherry-Palladin-C-7 and GFP-M in A549 cells (Fig. 3B). mCherry-Palladin-C-7 was detected at 100 kDa when co-transfected with GFP-M, as well as with GFP (panel labeled mCherry-Palladin-C-7). Both controls, mCherry-C1 and GFP, were detected at expected sizes, 28 kDa and 27 kDa, respectively (panels labeled mCherry and GFP). In addition, GFP-M was detected at the expected size of 55 kDa when co-transfected with mCherry-Palladin-C-7, as well as with mCherry-C1 (panel labeled GFP-M).

Interaction of M and palladin was also investigated in RSV-infected cells in a co-immunoprecipitation assay where anti-M antibody was used for immunoprecipitation, followed by Western blotting for palladin (Fig. 3C). Palladin (140 kDa) was detected in the unbound (UB) fractions only in the presence of anti-M antibody; significantly, the intensity of the palladin band increased in a dose-dependent fashion with increasing antibody to M (panel labeled 140 kDa). Palladin was neither detected in the absence of the primary antibody nor in any of the bound (B) fractions with or without anti-M antibody. A palladin specific band at 37 kDa was co-precipitated with M protein in a dose-dependent manner, correlating with increasing antibody to M (panel labeled 37 kDa). Our data demonstrates that M can bind two isoforms of palladin. Therefore, we hypothesize that a complex between M and two isoforms of palladin is formed, a larger isoform (140 kDa) and a smaller isoform (37 kDa), and that with increasing primary anti-M antibody, the higher molecular weight palladin dissociates from the complex containing the lower molecular weight palladin and M (Fig. 3D).

### GFP-M is retained in the cytoplasm upon microfilament destabilization when co-expressed with mCherry-Palladin-C-7

**Fig 4:**
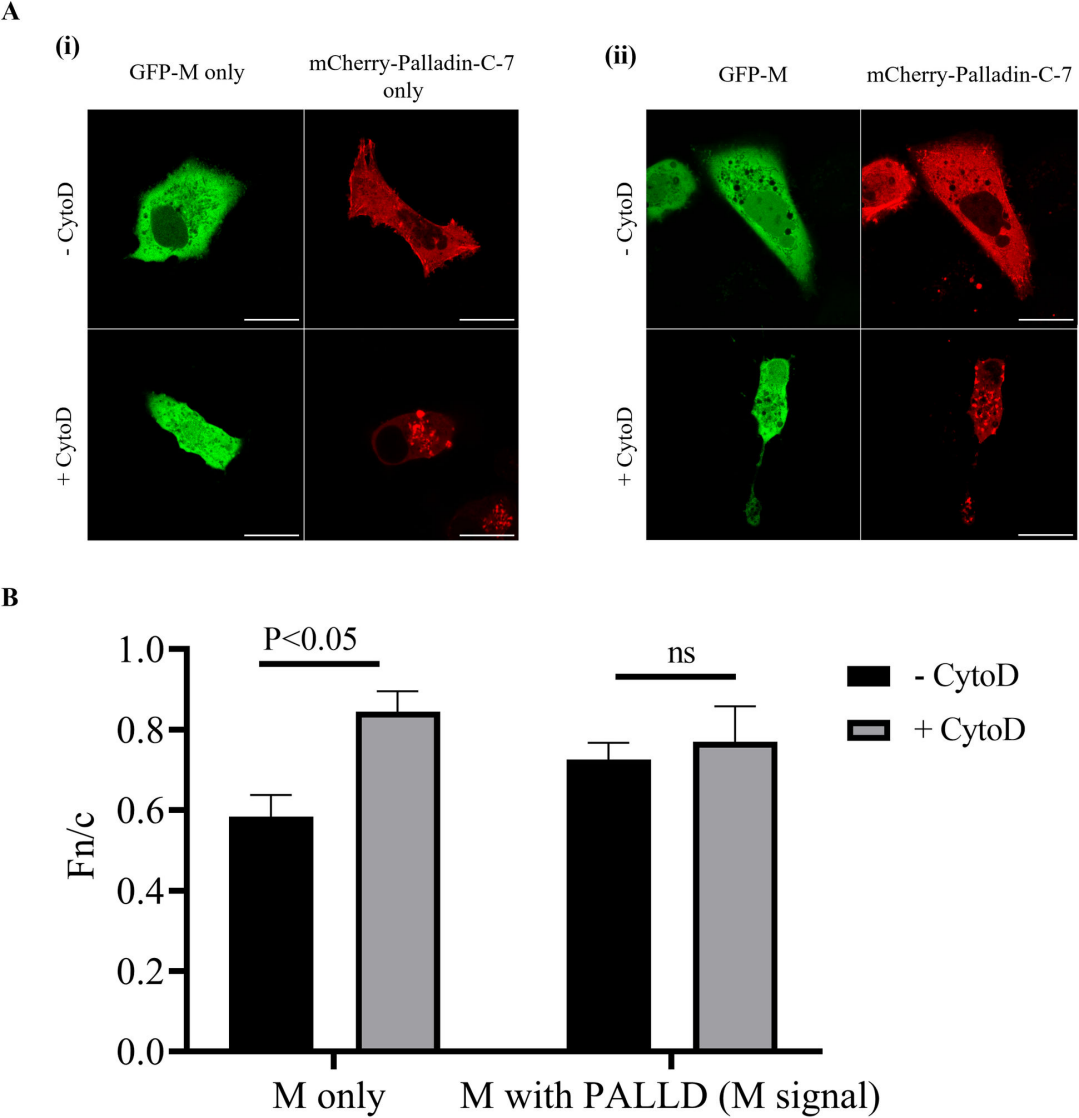
Overexpression of mCherry-Palladin-C-7 impacts M localization. (**A**) A549 cells were cultured overnight and transfected to express full-length GFP-M and mCherry-Palladin-C-7 either alone (i) or together (ii). Cells were left untreated (-CytoD) or treated with cytochalasin D (+CytoD) and imaged live using CLSM. Representative images are shown. Scale bars = 10 µm. Data shown are representative of three independent experiments. (**B**) Images such as those shown in (**A**) were analyzed by ImageJ to determine Fn/c. Data shown are mean ± SEM, ≥ 11. Statistically significant differences between groups are shown, ns = non-significant.

We have shown previously that M protein localizes to the nucleus on chemical destabilization of the microfilament network (4), suggesting that a microfilament-associated factor retains M in the cytoplasm. It is possible that this factor is palladin. To determine if this was indeed the case, the effect of cytoskeletal disruption on the subcellular localization of GFP-M and mCherry-Palladin-C-7 was investigated and visualized using live cell microscopy. As shown in Fig. 4A (i) (image labeled GFP-M, -CytoD), GFP-M was mainly cytoplasmic in A549 cells in the absence of cytochalasin D, as expected and as previously observed in COS-7 cells (4), and became diffused across the cell upon treatment with cytochalasin D (image labeled GFP-M, +CytoD). mCherry-Palladin-C-7 was mainly cytoplasmic in the absence of treatment when expressed alone (image labeled mCherry-Palladin-C-7, -CytoD) and remained cytoplasmic on treatment with CytoD (image labeled mCherry-Palladin-C-7, +CytoD). However, there was a change in its cytoplasmic localization, being present as punctate dots clustering close to the nucleus as opposed to being spread across the cytoplasm, showing association with microfilaments (compare images labeled -CytoD and +CytoD). The localization of mCherry-Palladin-C7 in the absence of CytoD was different to that depicted in Fig. 2, where it was both cytoplasmic and nuclear. The difference between the localization can be attributed to the different experimental conditions; in Fig. 4, the cells were imaged live for the visualization of subcellular localization, while in Fig. 2, the cells were fixed prior to imaging to visualize associations with the microfilament network. Figure 2 depicts the colocalization of mCherry–palladin with microfilaments after extraction of the nuclear and cytosolic material, which required fixation prior to probing with SiR Actin. In contrast, Fig. 4 depicts the localization of GFP-M and mCherry–palladin in live cells, with the fluorescent tags being able to be imaged without fixation as they require no further probing. It is known that fixatives can have varying effects on protein localization. As such, formaldehyde fixation can lead to redistribution of proteins due to the change in conformation of proteins. A method that can perfectly preserve the localization of a specific protein is currently unavailable (25). The use of formaldehyde in this study is dictated by its effects on the localization of M protein and suitability for the antibodies used (26).

Importantly, GFP-M was diffused across the whole cell when co-expressed with mCherry-Palladin-C-7, with no change in localization observed upon treatment (Fig. 4A (ii), images labeled GFP-M, +CytoD). There was no change in the localization of mCherry-Palladin-C-7 when co-expressed with GFP-M, regardless of treatment (compare images labeled mCherry-Palladin-C7 only and mCherry-Palladin-C-7). Results were confirmed with image analysis to determine the nuclear to cytoplasmic ratio (Fn/c) of GFP-M upon treatment with cytochalasin D (Fig. 4B); a value above 1 demonstrates movement into the nucleus. The localization of GFP-M was changed significantly with CytoD treatment when it was expressed alone (columns labeled M only), becoming close to equally distributed between the nuclear and cytoplasmic compartments (Fn/c = 0.82). Co-expression with mCherry-Palladin-C-7 resulted in some GFP-M being distributed to the nucleus (compare columns labeled -CytoD); this did not change in CytoD treatment (columns labeled M with PALLD). The data presented in Fig. 4 suggest that subcellular localization of GFP-M is influenced by mCherry-Palladin-C-7. If this is true, then a lack of palladin, e.g., by silencing, in RSV-infected cells would be expected to result in changed localization of M.

### Palladin silencing affects virus titer but not the subcellular localization of RSV M

Palladin siRNA reduced the mRNA levels of palladin in A549 cells as determined by real-time quantitative PCR and Western blotting. Approximately greater than 95% downregulation of mRNA and a −5.15-fold-change in expression was observed.

**Fig 5:**
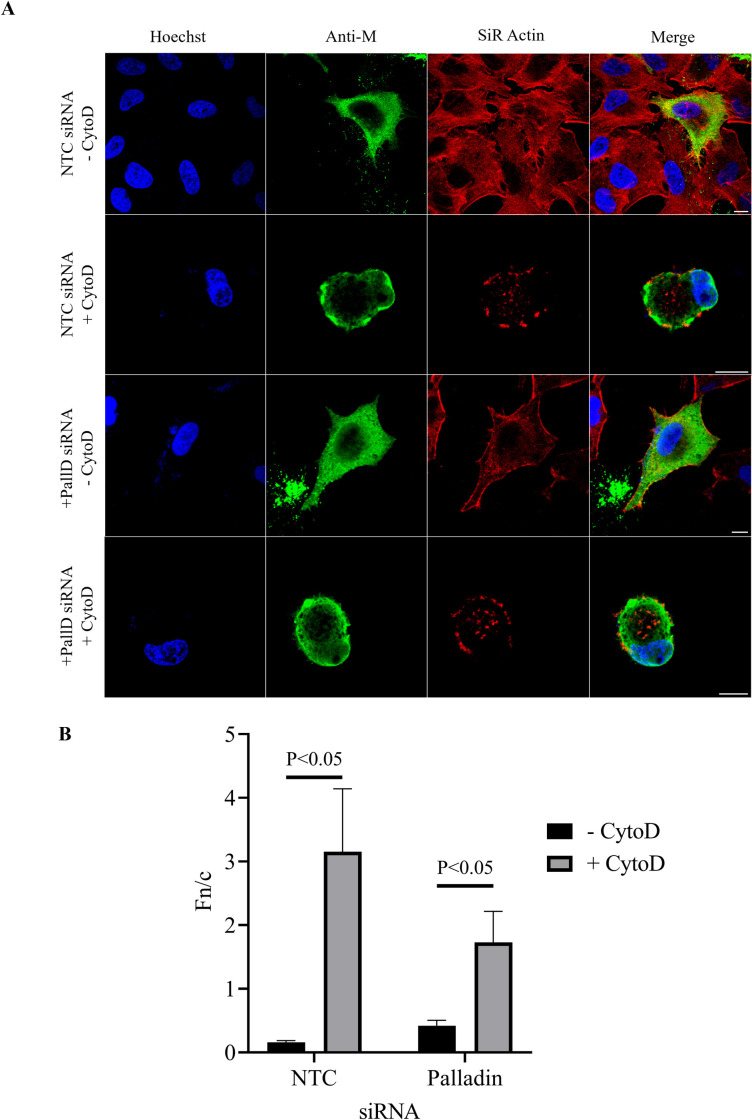
Palladin silencing affects M subcellular localization. (**A**) A549 cells were reverse transfected with palladin siRNA for 24 h before infection with RSV A2 for a further 24 h and were left untreated (-CytoD) or treated with cytochalasin D (+CytoD) for 12 h. Cells were fixed and probed for the visualization of the microfilament network (SiR Actin; red), RSV M (green) and the nucleus (Hoechst; blue). Scale bars = 10 µm. (**B**) Images such as those shown in (**A**) were analyzed by ImageJ to determine Fn/c for M protein. Data shown are mean ± SEM, ≥ 15. Statistically significant differences between groups are shown. Data shown are representative of two independent experiments.

There was no effect on the subcellular localization of RSV M in infected cells upon palladin silencing, in cells treated with cytochalasin D (Fig. 5) when compared with control (transfected with NTC siRNA) cells. RSV M (in green) was observed to be mainly cytoplasmic in untreated palladin-silenced cells (Fig. 5A, labeled +PallD siRNA -CytoD). Upon cytochalasin D treatment, RSV M was observed to localize in the nucleus in the presence of palladin siRNA (Fig. 5A, labeled +PallD siRNA +CytoD). Similarly, RSV M was observed to be mainly cytoplasmic when left untreated in non-targeting control cells (Fig. 5A, labeled NTC siRNA -CytoD), localizing to the nucleus upon cytochalasin D treatment (Fig. 5A, labeled NTC siRNA +CytoD). In addition, the microfilament network (in red) remained intact without treatment with cytochalasin D regardless of the presence of palladin siRNA (Fig. 5A, labeled -CytoD SiR Actin). Upon cytochalasin D treatment, the microfilament network collapsed, regardless of palladin silencing (Fig. 5A, labeled +CytoD SiR Actin).

Results were confirmed with image analysis to determine the nuclear to cytoplasmic ratio (Fn/c) of RSV M upon palladin silencing and treatment with cytochalasin D (Fig. 5B). The Fn/c analysis demonstrated that RSV M became nuclear in the presence of cytochalasin D regardless of palladin silencing (labeled +CytoD, compare NTC and palladin). However, levels of M in the cytoplasm without treatment and silencing are minimal compared with silenced cells (compare NTC and palladin -CytoD), demonstrating that without palladin, there is more M in the cytoplasm. In addition, the levels of M are higher in the nucleus in treated unsilenced cells compared with the palladin-silenced cells (compare NTC and palladin +CytoD), suggesting that not all of M is nuclear in the absence of palladin. These results demonstrate that palladin may influence the subcellular localization of M as there was a change in the degree of localization upon palladin silencing. The difference observed in the localization in Fig. 4 and 5 can be attributed to the different experimental conditions; in Fig. 4, the cells were imaged live to visualize subcellular localization in transfected cells, while in Fig. 5, the cells were fixed with formaldehyde prior to imaging to allow staining for the M protein and the microfilament network in RSV infected cells. As mentioned previously, fluorescent tags, such as those used in transfected cells depicted in Fig. 4, are able to be imaged without fixation as they require no further probing, while cells as depicted in Fig. 5, required fixation to allow probing with SiR Actin and antibody to RSV M. As described previously, fixatives can affect the subcellular localization of proteins, and this includes the relative presence in the nucleus and cytoplasm (25).

**Fig 6:**
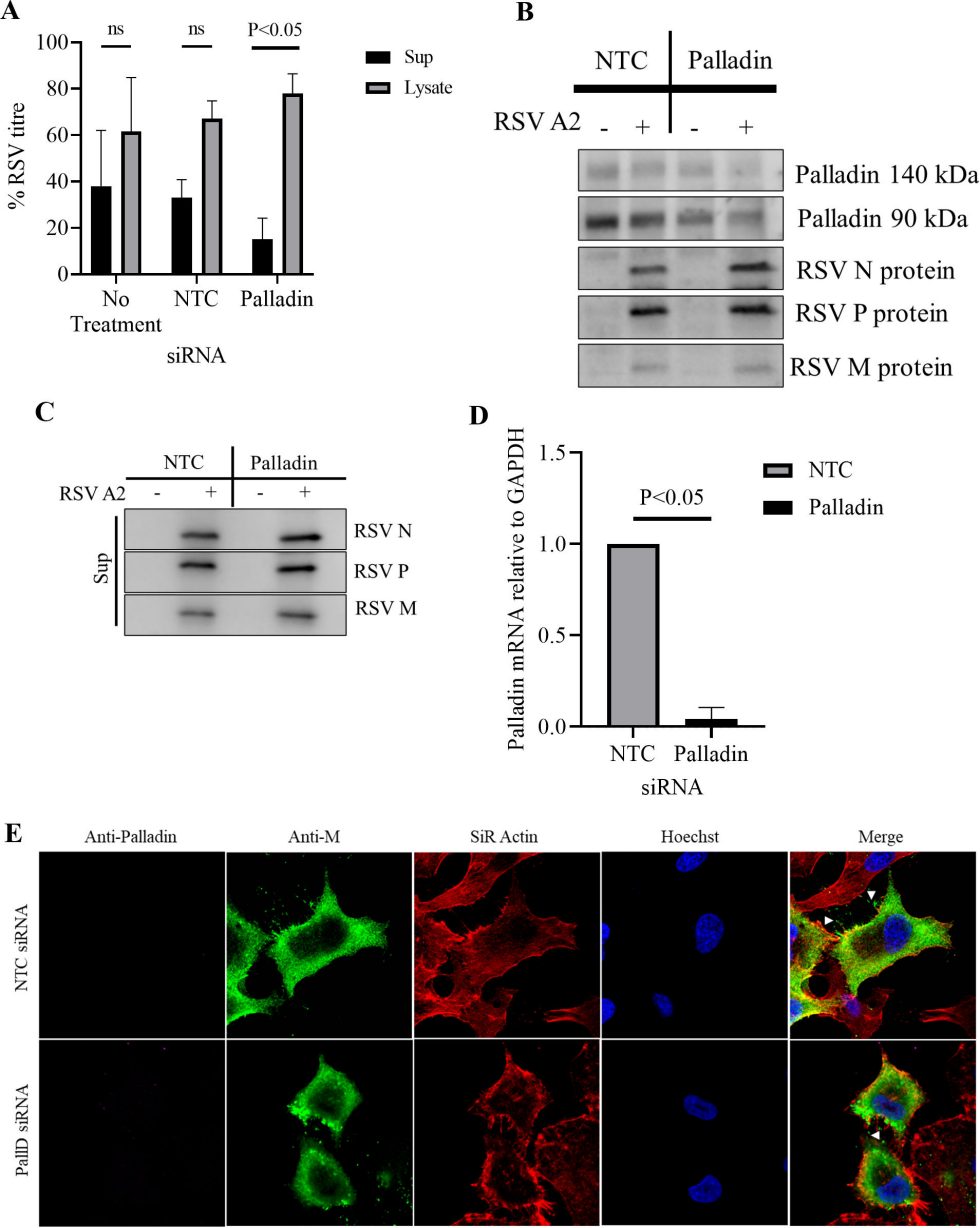
Palladin silencing affects RSV release. A549 cells were mock transfected (labeled No Treatment) or reverse transfected with palladin siRNA or a non-targeting control (labeled NTC) for 24 h. Cells were either left uninfected or infected with RSV at an MOI of 1, and supernatants or lysates were collected at 48 h.p.i. Data shown are representative of two independent experiments. (**A**) Supernatant and lysate samples were collected at 48 h.p.i., and viral titer (pfu/mL) was determined by immunoplaque assay. Data are mean ± SD. Statistically significant differences between groups are shown, ns = non-significant. (**B**) Lysates were collected and analyzed by Western blot. Membranes were probed for palladin (panels labeled 140 and 90 kDa) and RSV proteins (panel labeled RSV N, RSV P, and RSV M). Full Western blot images are provided as supplemental material (Fig. S4). (**C**) Supernatants were collected and used in a sucrose cushion virus-budding assay, and purified virus was analyzed by Western blot. Membranes were probed for the detection of RSV proteins (N, P, and M). Data shown are representative of two experiments. Full Western blot images are provided as supplemental material (Fig. S5). (**D**) RNA was extracted and reverse transcribed, and cDNA was analyzed by real-time quantitative PCR. Data shown are representative of two experiments. Data are mean ± SD. Statistically significant differences between groups are shown, ns = non-significant. (**E**) A549 cells were reverse transfected with palladin siRNA for 24 h before infection with RSV A2 for a further 24 h, fixed and probed for the visualization of the microfilament network (SiR Actin; red), palladin (magenta), RSV M (green), and the nucleus (Hoechst; blue) and viewed using CLSM. Colocalization is shown in yellow in merged images. White arrows represent RSV M external to the cell. Data shown are representative of two independent experiments.

In the absence of siRNA, the RSV titer in the supernatant (released RSV) was similar to that in the lysate (cell-associated RSV; Fig. 6A, labeled no treatment). This was also observed in cells that had been transfected with the non-targeting control (Fig. 6A, labeled NTC). Upon palladin silencing, the virus titer in the supernatant (labeled palladin, sup) was significantly lower than that in the lysate (labeled palladin, lysate). Clearly, reducing the amount of palladin affects RSV budding. This is not due to reduced viral protein expression as silencing did not reduce expression of RSV proteins N, P, and M (Fig. 6B). Interestingly, the N, P and M protein levels appeared to increase in the supernatant of cells after palladin silencing (Fig. 6C, compare the protein bands in lanes labeled NTC + and Palladin+). Palladin silencing was effective, with expression of both 140 and 90 kDa isoforms of palladin being reduced (Fig. 6B), correlating with a significant reduction in palladin mRNA (Fig. 6D). In addition, RSV M and actin microfilaments colocalize (Fig. 6E, upper panel, labeled merge, shown in yellow) at the cell periphery at the foot of and along the SiR Actin-labeled filopodia. RSV M is also observed external to the infected cell when in proximity with a nearby cell and can be seen emerging from the filopodia to join with the filopodia of the adjacent cell (Fig. 6E, upper panel, white arrows). However, upon palladin silencing, RSV M is observed to be concentrated near the foot of the filopodia instead of along it and no colocalization with actin is observed (Fig. 6E, lower panel, labeled Merge). While RSV M is observed external, incidence is reduced and cannot be seen in connecting filopodia (Fig. 6E, lower panel, white arrow). Taken together, our data suggest that palladin may play a role in viral fitness, and its silencing may produce non-infectious RSV progeny.

## DISCUSSION

In the current study, we have shown that RSV M can associate with microfilaments, and that palladin facilitates this interaction through a complex interaction with RSV M. Additionally, we have shown that palladin downregulation results in reduced virus release, suggesting an important role for palladin in RSV release. As a dynamic ABP (27), palladin’s ability to interact with microfilaments provides it with potential to mediate the movement of M and vRNPs to the cell surface.

Previously, we showed an association between M and microfilaments in cell culture (4). This was shown by CLSM and by inference from the fact that chemical destabilization of microfilaments results in mis-localization of M in transfected cells. As microfilaments are frequently nucleated at the plasma membrane, they are located in the highest concentration at the cell periphery (28). RSV assembly sites are also located at the plasma membrane, leading to the proposed hypothesis that the transportation of vRNPs to these sites could involve the microfilament network (3).

Here, we show that RSV M colocalizes with microfilaments in both RSV-infected and GFP-M-transfected cells (Fig. 1, images labeled unextracted). With the removal of nuclear and cytosolic proteins in RSV-infected cells, an M-containing complex was observed to be tethered to thin, as opposed to thick microfilaments in both Vero and A549 cells. The data presented concur with the previous finding by Ulloa et al. (2) that a complex containing the RSV viral proteins N, P, M2-1, plus M is anchored onto the microfilament network (2). As the M-containing complex was observed to associate with the thinner actin filaments in the actin cell cortex, the meshwork underlying the plasma membrane (29), and not directly with filaments at the membrane, the data presented further support that M plays a role in transporting RNPs to assembly sites. However, further research is required to confirm the exact viral constituents of the M-containing complex observed in this study.

M also colocalized with microfilaments in cells transfected to express GFP-M. Interestingly, upon removal of the nuclear and cytosolic proteins, GFP-M localized toward the microfilaments without direct association. While the findings of the current study show that there is no direct association in cell culture, M has been shown to interact with actin directly *in vitro* (4). Many ABPs have the ability to bind proteins that bind actin (6) and can act as scaffolding proteins to recruit such proteins to the microfilament network (7). This prompted the investigation into the facilitation of the M–actin interaction by ABPs.

We found an interaction between the ABP palladin and RSV M. Palladin has been shown to colocalize with several ABPs and also with actin-rich structures (19). Our data show that palladin colocalizes with M (Fig. 2A) and actin filaments (Fig. 2B). M and palladin colocalized throughout the cell when left untreated, colocalizing towards microfilaments on extraction of nuclear and cytoplasmic proteins (Fig. 2A, bottom row, labeled cytosolic and nuclear proteins extracted), as expected (4). The colocalization of M and palladin towards microfilaments (Fig. 2A, bottom row, labeled cytosolic and nuclear proteins extracted), suggest that M is indeed associating with microfilaments indirectly through its interaction with palladin. The mechanism through which M is recruited to microfilaments by palladin is unclear and requires further investigation.

We found that RSV M associates with two isoforms of palladin, 140 and 37 kDa, in RSV infected cells (Fig. 3C). Palladin 37 kDa was shown to form a complex with M, while the 140 kDa palladin was detected in the unbound fraction in co-immunoprecipitation studies. The 140 kDa palladin increased with increasing amounts of anti-M antibody. We hypothesize that with increasing amounts of anti-M antibody, the M and 37 kDa palladin dissociate from the 140 kDa palladin and are pulled down. As a result, the 140 kDa palladin is released into the unbound fraction, while the palladin 37 kDa bound to M remains in the bound fractions. The M–palladin interaction was confirmed in transfected cells, where mCherry-Palladin-C-7 and GFP-M co-precipitated (Fig. 3A). The mCherry-Palladin-C-7 construct contains ~72 kDa of the C-terminal sequence of palladin, which includes the section postulated to be included in the palladin 37 kDa and the palladin 140 kDa.

The molecular weights of only three of the nine predicted isoforms of human palladin have been characterized to date (90, 140, and 200 kDa) (18, 30–32). The molecular weights of the minor isoforms have only been predicted, and human cells may express palladin variants that are not detected by all antibodies (18, 32). Ours is the first study to demonstrate the presence of a 37 kDa palladin isoform. The identity of this particular isoform will need to be confirmed by proteomic techniques. Future advancement in the characterization of the different palladin isoforms will assist in elucidating the M and palladin interaction.

Previously, we have shown that the M protein localizes to the nucleus upon chemical destabilization of the microfilament network (4), suggesting that M may require the actin cytoskeleton for movement out of the nucleus. As M associates with microfilaments through interactions with palladin (current study), it is possible that palladin may mediate the M–actin interaction and cytoplasmic retention of M following its movement out of the nucleus. One study has shown that palladin localizes to the nucleus from the cytoplasm in Sertoli cells (33). Another study has shown that palladin can re-localize to the cytoplasm from the nucleus upon cytoskeletal destabilization (34). The findings presented in the current study show that cytoskeletal destabilization does not affect the subcellular localization of palladin in A549 cells, which remains unchanged regardless of co-expression with GFP-M (Fig. 4).

Interestingly, when co-expressed with palladin, actin destabilization did not affect M subcellular localization; M remained both cytoplasmic and nuclear regardless (Fig. 4). Palladin silencing had no effect on the subcellular localization of RSV M (Fig. 5) as M became nuclear upon cytoskeletal destabilization, as shown previously (4). However, while results demonstrated movement into the nucleus, palladin silencing resulted in more M being retained in the cytoplasm with less localized to the nucleus compared with the NTC samples. Taken together, these results, and those shown in Fig. 4, demonstrate that palladin may influence the subcellular localization of M to some extent maybe stabilizing the M–actin interaction. Indeed, palladin is known as a stabilizing protein, with a previous study showing that palladin plays a role in stabilizing actin filaments, as well as the transmembrane linker β1 integrin, a cell surface protein that attaches the cell cytoskeleton to the extracellular matrix (35) functioning to connect filopodia to surrounding cells (36).

Filopodia are actin-rich protrusions of the membrane that play a role in sensing the environment to make stable contacts during cell spread (36–38). A key player involved in the initiation of filopodia is the nucleator of branched F-actin filaments, the Arp2/3 complex. Arp2/3 facilitates the reorganization and assembly of the actin network that subsequently forms filopodia (37). Indeed, filopodia play a role in shuttling RSV particles to nearby cells, and the depletion of Arp2 has been shown to reduce virus production, filopodia formation, cell motility, and viral spread (38, 39). In addition, Arp2 knockdown in RSV infection has been shown to cause the disorganization of virus filaments at the cell surface (39). Although a role for the RSV F protein in initiating the formation of filopodia to prepare the cell for spread has been identified, further studies are required to determine if other RSV proteins, including M, are also involved. Interestingly, palladin localizes to filopodia with other actin-organizing proteins (40) and can effectively replace the Arp2/3 complex during *Listeria monocytogenes* infection (41).

Although palladin and Arp2/3 have overlapping roles in actin dynamics, there is no direct association between them, and their potential compensatory roles in RSV filopodia formation are yet to be determined. While filopodia have a role in migration, albeit not alone, they mainly function to establish cell-to-cell contact (36), which is the case in RSV infection (39).

Importantly, palladin silencing resulted in reduced viral titer in released virus and increase in the cell-associated virus in infected cells (Fig. 6A), suggesting a role for palladin in RSV release. The observed changes in viral titer were not a result of reduced viral protein expression (Fig. 6B), but probably due to the reduced replication fitness of the released virus. RSV M was concentrated at the foot of and not along filopodia in palladin-silenced infected cells (Fig. 6E, lower panel), in contrast to the NTC-treated cells, where M colocalizes with actin at the cell periphery and throughout the filopodia (Fig. 6E, upper panel, labeled merge, shown in yellow). We have shown previously that ordered distribution of M along the whole filamentous virus is required for RSV replication fitness (42). Intriguingly, RSV M could be seen external to the cell appearing to originate from the filopodia of one cell and join with that of the other (Fig. 6E, upper panel, labeled merged, white arrows). This was not observed in palladin-silenced cells despite some RSV M seen external to the cell (Fig. 6E, lower panel, labeled marge, white arrow). Interestingly, our findings mirror those of a previous study where RSV F protein was shown to colocalize with actin at the cell periphery and along filopodia extending from one cell and joining with that of another (38). Clearly, filopodia formation and utilization is important for the cell-to-cell spread of RSV, and our data suggest the involvement of palladin in the process.

RhoA, a member of the Rho GTPase family and organizer of the actin microfilament network, has been implicated in RSV infection, and its interaction with RSV F protein is important for the formation of filamentous RSV (43–45). As previous studies have shown the involvement of RhoA in syncytium formation through its inhibition, a role for RhoA in RSV viral budding has been suggested (46). Interestingly, previous studies have shown an association between palladin and RhoA, with a role in cytoskeletal organization (19, 47). Additionally, Arp2/3 can regulate RhoA activity and protein levels, with Arp2/3 depletion leading to increase in RhoA activity (38, 48).

**Fig 7:**
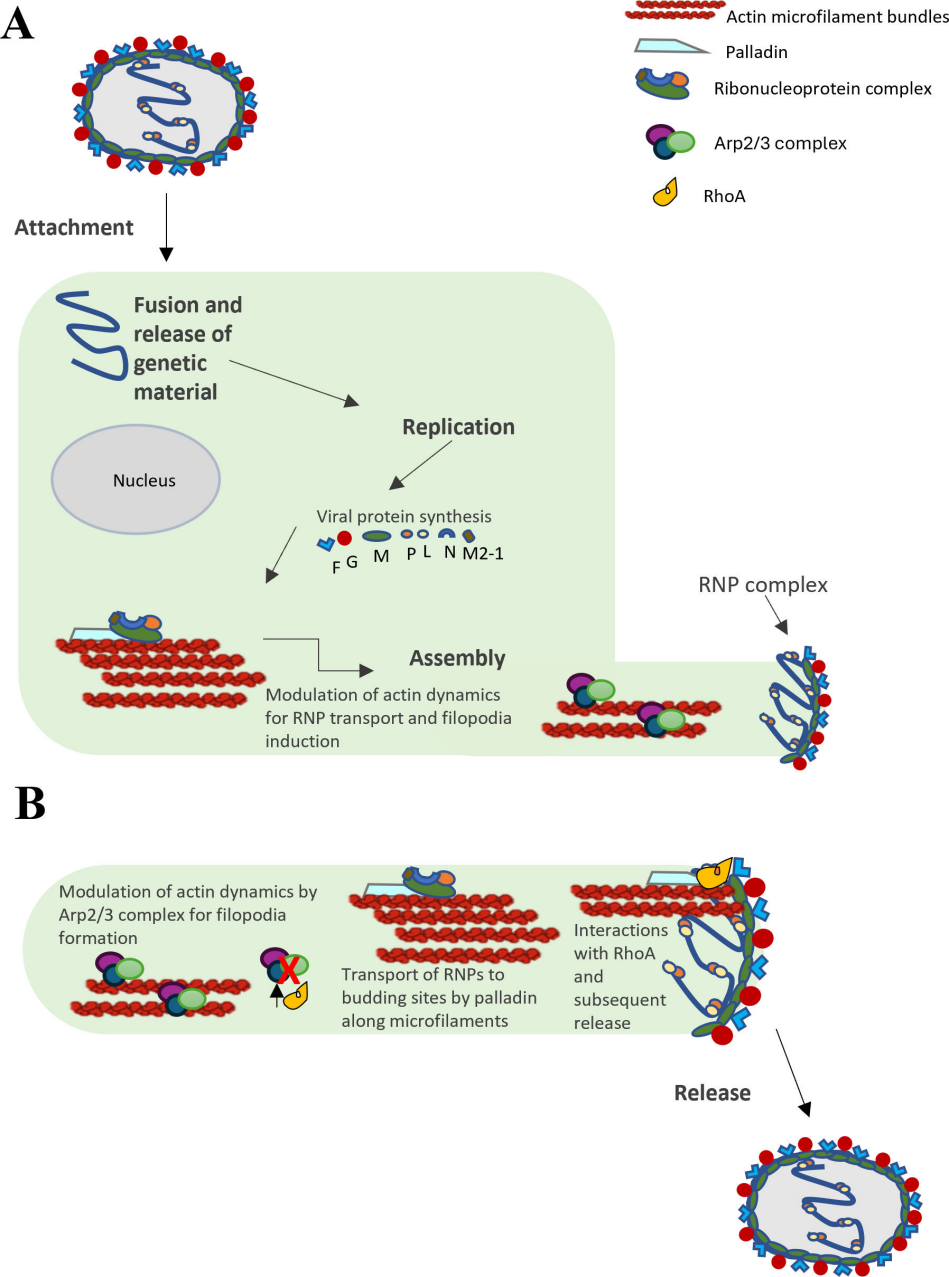
Model. Transport of M-RNPs to assembly and budding sites by palladin. (**A**) The RSV life cycle begins with attachment to the host cell to initiate entry by fusion before release of genetic material into the cytoplasm. The viral genome is then replicated, and viral proteins are synthesized. Formation of the ribonucleoprotein (RNP) complexes consisting of the nucleoprotein (N), large polymerase protein (L), phosphoprotein (P), and the M2-1 protein takes place, which are then transported to the plasma membrane by the matrix (M) protein. Palladin facilitates the transport of M-RNPs along microfilaments to sites of assembly before the initiation of budding processes. Arp2/3 complex and palladin work together to modulate actin dynamics to enable transportation and initiate filopodia induction and formation. (**B**) Formation of filopodia by Arp2/3 is followed by the inactivation of the Arp2/3 complex, leading to increase in RhoA activity. Palladin continues actin dynamic modulation to move M-RNPs to filopodia budding sites where it interacts with RhoA to initiate budding and release of mature virions.

As previous reports have shown a reduction in RSV release upon Arp2/3 silencing, and our data demonstrates a similar reduction upon palladin silencing, without complete inhibition, we hypothesize that palladin and the Arp2/3 complex work together to enable the bundling–debundling of actin microfilaments that enable movement of the M–RNP complex to the cell surface (Fig. 7). The Arp2/3 complex may specifically be involved in actin nucleation for RSV filopodia induction and formation, and regulation of RhoA activity (Fig. 7A). Palladin continues dynamic modulation of actin, moving M-RNPs to the filopodia budding sites at the plasma membrane, where it associates with RhoA (Fig. 7B). The final release of mature virions is through an as yet unknown mechanism that could likely involve RhoA and its possible interactions with palladin. As such, further investigation into the role of palladin and its interaction with RSV M and RhoA in budding will provide a deeper understanding of RSV budding processes.

In conclusion, while palladin has been investigated in diseases including kidney disease, cardiovascular disease, and cancer (i.e. pancreatic ductal adenocarcinoma; colorectal cancer) (32, 49–51), this study is the first to report a role for palladin in the infection and replication of a major respiratory pathogen and its association with a viral protein.

### Limitations of the study

A limitation of this study is its use of overexpression systems. Overexpression systems have the potential to produce artefactual results that may not mimic the true function of the proteins and their interactions (52, 53). This could be a consequence of stoichiometric imbalance, promiscuous interactions, resource overload, or pathway modulation; the occurrence of these mechanisms is dependent on the degree of overexpression (53, 54). The use of overexpression systems in this study was important, as there is a lack of high-quality antibodies to study M and palladin in the context of the infected cell. However, it is important to note that despite relying on overexpression systems, we also show that without overexpression, including with silencing, palladin plays a role in RSV infection and, importantly, can indeed associate with RSV M.

## MATERIALS AND METHODS

### Cells and viruses

Human type II respiratory epithelial (A549) cells and African Green Monkey Kidney (Vero) cells were obtained from the European Collection of Authenticated Cell Cultures (ECACC). Cells were maintained in high glucose Dulbecco’s modified Eagle’s medium (DMEM; Sigma-Aldrich Pty Ltd.) with 10% heat-inactivated fetal bovine serum (FBS; Interpath) supplemented with penicillin, streptomycin, and neomycin (PSN; Life Technologies/ThermoFisher Scientific) at 37°C in 5% CO_2_.

RSV subtype A2 strain obtained from the American Type Culture Collection (ATCC) was used for all virus infections and grown in Vero cells, maintained as above in DMEM with 2% FBS and PSN. Virus was harvested when 50% cytopathic effect (CPE) was observed. Culture supernatant was collected, and cells were lysed in serum-free medium containing SPGA (218 mM sucrose, 7.1 mM K2HPO4, 4.9 mM sodium glutamate, 1% (wt/vol) bovine serum albumin). Harvested supernatant and lysate were clarified by centrifugation at 3,893×*g* for 15 min at 4°C and stored at −80°C for lysis. RSV titer was determined by immuno-plaque assay and viral plaque-forming units per mL (PFU/mL) calculated as previously described (4).

### Plasmids used in this study

The mammalian cell expression constructs for GFP and GFP-M (1-256) have been described previously (55). The mCherry-Palladin-C-7 plasmid was a gift from Michael Davidson (Addgene plasmid # 55113; https://n2t.net/addgene:55113; RRID: Addgene_55113).

### Lipofectamine 2000 transfection

Overnight sub-confluent monolayers of A549 or Vero cells were transfected to express proteins of interest using Lipofectamine 2000 (Invitrogen; 1:1 mix of DNA and reagent) and incubated at 37°C with 5% CO_2_ for 18–24 h. For subcellular localization experiments, cytochalasin D was added at 18 h post-transfection (p.t.) for 6 h. Cells were visualized by confocal laser scanning microscopy (CLSM); see below.

### Cell lysate preparations

Cells were lysed and prepared for Western blot analysis by washing once with ice-cold 1× phosphate buffered saline (PBS) before lysis with RIPA buffer (150 mM NaCl, 1% Triton-X, 0.5% sodium deoxycholate, 0.1% SDS, 50 mM Tris-HCl, 1× protease inhibitor, 1× phosphatase inhibitor). The cells were incubated on ice with constant agitation for 30 min, collected, and centrifuged to pellet cellular debris at 13,523×*g* at 4°C for 20 mins. Clarified supernatants were transferred to fresh pre-cooled tubes and stored at −80°C until required.

### Native cytoskeleton enrichment

Vero or A549 cells grown overnight on glass coverslips in 12-well plates were either left uninfected or infected with RSV A2, transfected to express GFP-M, or co-transfected to express GFP-M and mCherry-Palladin-C-7. To evaluate microfilament enrichment, A549 cells were mock transfected as described previously. For RSV infection, the cells were infected at a multiplicity of infection (MOI) of 1 in DMEM with 2% FBS supplemented with PSN. The virus inoculum was replaced with fresh medium after 1 h of adsorption, and this time point is referred to as “0” h. A native cytoskeleton enrichment was undertaken at 24 h post-infection (p.i.) or p.t. using the ‘Proteoextract Native Cytoskeleton Enrichment and Staining Kit’ (Merck), as per manufacturer’s recommendations. The microfilament network was visualized by staining cells with either Alexa Fluor 568 phalloidin (Invitrogen; 1:100 dilution for Vero cells) or SiR Actin (Cytoskeleton, Inc.; 1:1000 for A549 cells). A549 cells mock transfected were stained for GAPDH (Merck) to evaluate microfilament enrichment; effective enrichment is demonstrated by no GAPDH signal in extracted cells. Coverslips were mounted on glass slides with DAKO fluorescent mounting media (Agilent), and slides were incubated at room temperature for 20 min in the dark to allow maturation. The cells were visualized by CLSM or stimulated emission depletion (STED) microscopy; see below.

### Immunofluorescence assay

Fixed, permeabilized cells on coverslips were immuno-stained with primary antibodies specific to the protein of interest for 30 min at room temperature. The cells were washed with PBS, incubated for 30 min at room temperature in species-specific secondary antibody-conjugated Alexa Fluor fluorescent dyes (Invitrogen; 1:1,000). The cells were washed before incubation in 0.5 µL/mL Hoechst-33432 (nuclear counterstain; Invitrogen) for 5 min, washed once again, and coverslips mounted on glass slides with DAKO fluorescent mounting media as above. Images were acquired by CLSM as below.

### Microscopy

#### Confocal microscopy

Fixed (transfected and RSV infected) cells were viewed by confocal microscopy as previously described (4). Briefly, the cells were viewed using the 60× oil immersion objective lens on the Nikon Eclipse Ti confocal laser scanning microscope (CLSM) (Nikon Instruments Inc.), and images were acquired using the NIS Elements software (Version 4.0, Nikon Instruments Inc.). For live cell microscopy, FluoroBrite DMEM (Gibco) was added to coverslips in wells and imaged as above. Additionally, live (siRNA transfected) cells grown in plates with glass bottoms (Cellvis) were viewed under the 60× objective lens on the Nikon Eclipse Ti 2 CLSM with a stage top chamber (Okolab). The cells were incubated at 37°C, and images were acquired using the NIS Elements software (Version 5.30.02, Nikon Instruments Inc.). Cytoskeleton enrichment was evaluated using the Nikon Eclipse Ti 2 CLSM and corresponding software as above.

#### Stimulated emission depletion (STED) microscopy

Transfected and RSV-infected cells used in native cytoskeleton enrichment experiments were fixed, and super-resolution images were acquired at the Australian Centre for Microscopy & Microanalysis (ACMM) at the University of Sydney using the 100× oil immersion objective lens on the Leica TCS SP8 STED 3X microscope and corresponding Leica Application Suite X (LAS X, Leica Microsystems, version 3.7.0.20979) software. The deconvolution of the high-resolution images was undertaken by ACMM light microscopist Neftali Flores Rodriguez. Scale bar sizes were determined in ImageJ.

#### Colocalization analysis

To quantify colocalization between points in dual-color images, pixel intensity spatial correlation analysis was undertaken using ImageJ. Two colocalization coefficients were used to express the intensity correlation of colocalizing objects: Pearson’s correlation coefficient (PCC) and Manders’ coefficients (MCs; M1 and M2). The PCC is used to determine the overlap between two images by measuring their pixel-by-pixel signal covariance, normalized by the product of their standard deviations, assuming a linear relationship. The PCC values range from −1 to 1, as the calculation determines the extent the variation in signal intensity of one image can be explained by the signal variation in the corresponding image and, thus, involves the difference of pixel intensity from the population mean. A PCC value of 1 demonstrates that fluorescence intensities for the two images are perfectly linearly related, while a value of −1 indicates that the intensities are perfectly linearly unrelated. A PCC value of 0 indicates that the predictive value between the two images is limited, and, thus, a correlation does not exist between them (20, 21). Therefore, the PCC assumes that if the two targets are functionally related, their abundances will also be predictably related regardless of their location within a given region. The Manders’ coefficients (MCs; M1 and M2) are used to quantify the co-occurrence of two molecules of interest in a particular location within a cell and does not determine if a predictable relationship exists between the intensities in the two images. Therefore, the MCs are used to calculate what percentage of intensity from one channel overlaps the intensity from the other channel (22). This is undertaken to determine the degree of spatial overlap between the two structures present in the cell (21).

### M–actin interaction

Vero or A549 cells grown overnight were either infected with RSV A2 at MOI of 1 or transfected to express GFP-M, as previously described, before native cytoskeleton enrichment at 24 h p.i. and 18–20 h p.t., respectively. RSV-infected cells were stained for the visualization of M protein (anti-MB50; gift from Errling Norrby, Sweden) and microfilaments (Alexa Fluor 568 phalloidin, 1:100 dilution for Vero cells or SiR Actin, 1:1,000 for A549 cells), including Hoescht 33432 (0.5 µL/mL). Transfected cells were also stained with either Alexa Fluor 568 phalloidin (1:100; Vero cells) or SiR Actin (1:1000; A549 cells) for the visualization of the microfilament network.

### M interaction with the actin-binding protein palladin

A549 cells were grown overnight on glass coverslips in a 12-well plate and co-transfected to express GFP-M and mCherry-Palladin-C-7. Control cells received the same number of medium changes without plasmid DNA. At 24 h p.t., a native cytoskeleton enrichment was undertaken as described above. The cells were stained with SiR Actin to visualize the microfilament network. Confocal microscopy was undertaken as above, and scale bar sizes were determined using ImageJ. Colocalization between points in dual-color images were quantified by pixel intensity spatial correlation analysis using ImageJ as described previously.

### Co-immunoprecipitation

Overnight sub-confluent monolayers of A549 cells in six-well plates were either co-transfected or infected with RSV A2 at an MOI of 1 and lysed at 16–18 h p.i. with RIPA buffer, as described above. Primary antibody was added to lysates in increasing amounts and precipitated with EZview Red Protein G Affinity Gel (Sigma); control cells received the same treatment without the addition of primary antibody (0 µL). Following immunoprecipitation, unbound (UB) and bound (B) proteins were analyzed by Western blot.

Cells co-transfected to express mCherry-Palladin-C-7 and GFP-M were processed for fluorescence-activated cell sorting (FACS). At 18 h p.t., media were aspirated from wells, and cells were washed with pre-warmed PBS. Following the wash step, cells were harvested by trypsinization and diluted in 2% FBS in DMEM with PSN. The cell suspension was collected, kept on ice, and transported to the John Curtin School of Medical Research Imaging and Cytometry Facility at the Australian National University for cell sorting. FACS was used to sort cells into double positives (GFP-M and mCherry-Palladin-C-7 expressing cells). Sorted cells were centrifuged at 13,523×*g* for 10 min at 4°C, the supernatant was discarded, and the pellet washed once with ice-cold 1× PBS. The centrifugation was repeated, and the supernatant was discarded before the cells were lysed with RIPA buffer, and the co-immunoprecipitation was undertaken as described above. Anti-mCherry (Abcam) primary antibody was added to lysates and immunoprecipitated as above. Control samples had an equivalent volume of RIPA buffer added without antibody. Both UB and B fractions were analyzed by Western blot, as described previously, and membranes were probed for the presence of GFP.

RSV-infected cells were lysed, and co-immunoprecipitation was undertaken as described above. Anti-MB50 (gift from Errling Norrby, Sweden) primary antibody was added to lysates and immunoprecipitated as above, and both UB and B fractions were analyzed by Western blot. Membranes were probed for the presence of palladin (Abcam).

### Microfilament destabilization in cells transfected to express mCherry-Palladin-C-7 and GFP-M

Subconfluent monolayers of A549 cells were co-transfected to express GFP-M and mCherry-Palladin-C-7. At 18 h p.t., the cells were either left untreated or treated with cytochalasin D (4 µg/mL, Enzo Life Science) in serum-free medium for 6 h. The cells were visualized by live-cell microscopy as described above. Quantitative analysis was undertaken to determine the relative fluorescence of GFP-M and mCherry-Palladin in the nucleus to the cytoplasm (Fn/c) as previously described (4).

### Palladin siRNA experiments

Palladin siRNA (ON-TARGETplus Human PALLD 23022 siRNA; L-016891–00-0005) and a non-targeting control (NTC) (ON-TARGETplus Non-targeting siRNA; D-001910–01-05) were obtained from Dharmacon. A549 cells were washed with PBS, harvested by trypsinization, and resuspended in fresh medium shortly before transfection. Transfection complexes containing DharmaFECT transfection reagent and the siRNA were prepared as per manufacturer’s instructions as detailed in ‘DharmaFECT Transfection Reagents-siRNA transfection protocol’. A volume of the cell suspension was transferred to each well already containing the transfection complex. Palladin knockdown was confirmed by real-time quantitative PCR (RT-qPCR). Following RNA extraction (TRIzol Reagent User Guide Invitrogen), RNA transcription was undertaken using iScript Reverse Transcription Supermix (Bio-Rad). cDNA was then amplified by qPCR. Palladin primers (forward: CAGGCTGTCAACCAAAGAGGTC; reverse: TCGTCTCCACTGTCCCTTGATC; sequences provided by Origene) were synthesized by Sigma. GAPDH (Merck) was used as a housekeeping gene. The reaction was run on Applied Biosystems Real time qPCR machine (7500 Fast System SDS 21 CFR Part 11 Module; Invitrogen), and Ct data were collected and fold-change (Log2) in palladin expression was determined; there was a −5.15-fold-change in palladin expression with silencing.

In one experiment, transfections were allowed to proceed for 24 h before infection with RSV A2 strain at an MOI of 1 and incubated at 37°C with 5% CO_2_ for a further 48 h. Cells were harvested when 50% cytopathic effect (CPE) was observed. Supernatant and lysates were processed for titration as described above.

In another experiment, cells reverse transfected and infected as above, were treated with cytochalasin D at 24 h p.i., or left untreated for 12 h. Cells were fixed with 4% formaldehyde at 36 h p.i. (60 h p.t.) and visualized by CLSM, as described above. Quantitative analysis was undertaken to determine the relative fluorescence of RSV M in the nucleus to the cytoplasm (Fn/c) as previously described (4).

Additionally, A549 cells were reverse transfected with NTC and palladin siRNA and infected as above before supernatants were collected at 48 hrs p.i. Supernatants were clarified by centrifugation at 3,893×*g* for 15 mins at 4°C, transferred to fresh tubes, and stored at −80°C. The supernatants were used in a sucrose cushion virus budding assay as described below.

### Sucrose cushion virus-budding assay

Clarified RSV-infected supernatants were added to an ultracentrifuge tube containing ice-cold 30% sucrose in 1× PBS solution. The remainder of the ultracentrifuge tube was filled with 1× PBS before centrifugation at 20,000×*g* for 3 h at 4°C. Following centrifugation, the supernatant was discarded, and a volume of ice-cold 1× PBS was added to fill the tube, before being centrifuged once again for 1 h. The supernatant was discarded, and the pellet was lysed with a volume of RIPA buffer for 30 mins on ice with agitation. Following lysis, the lysate was centrifuged at 13,000×*g* for 30 min before supernatants were transferred to fresh tubes and analyzed by Western blot analysis to detect RSV proteins, as described previously.

## Data Availability

All generated data are provided within the article and the supplemental material.

## SUPPLEMENTAL MATERIAL

Supplemental figures jvi.01435-24-s0001.docx Figures S1 to S5.

ASM does not own the copyrights to Supplemental Material that may be linked to, or accessed through, an article. The authors have granted ASM a non-exclusive, world-wide license to publish the Supplemental Material files. Please contact the corresponding author directly for reuse.
