# Supplementary material for: The actin-binding protein palladin associates with the respiratory syncytial virus matrix protein
Source: J Virol. 2024 Oct 3;98(10):e01435-24. doi: 10.1128/jvi.01435-24 (PMC11494977; doi:10.1128/jvi.01435-24)
Supplement: Supplemental figures — Figures S1 to S5. [file jvi.01435-24-s0001.docx]

#
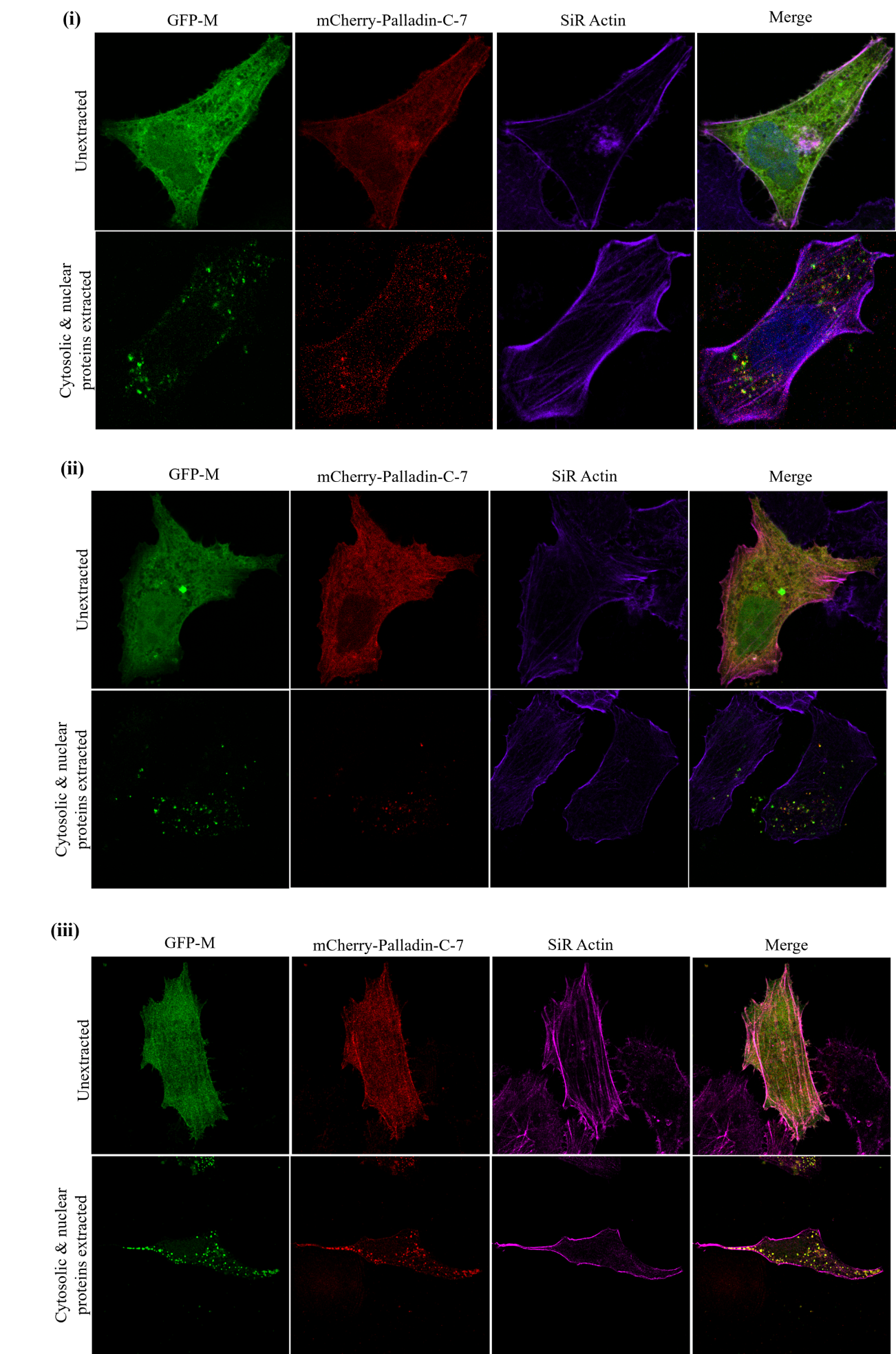


**Fig S1. Palladin associates and colocalizes with M and microfilaments in A549 cells.** Additional images for Fig 2. Cells were cultured overnight and transfected to express mCherry-Palladin-C-7 and GFP-M. Cytoskeleton enrichment was undertaken to determine the interaction between palladin and RSV M, and their interaction with the microfilament network. Transfected cells were either left untreated (images labelled unextracted) or treated (images labelled extracted) with enrichment buffers to remove soluble nuclear and cytosolic proteins. Cells were probed for visualization of the microfilament network (SiR Actin; in magenta). The colocalization of palladin (in red) and M (in green) is indicated in yellow (image labelled merge). **(i)** and **(ii)** Images were visualized by confocal microscopy. **(iii)** Images were visualized by STED microscopy.


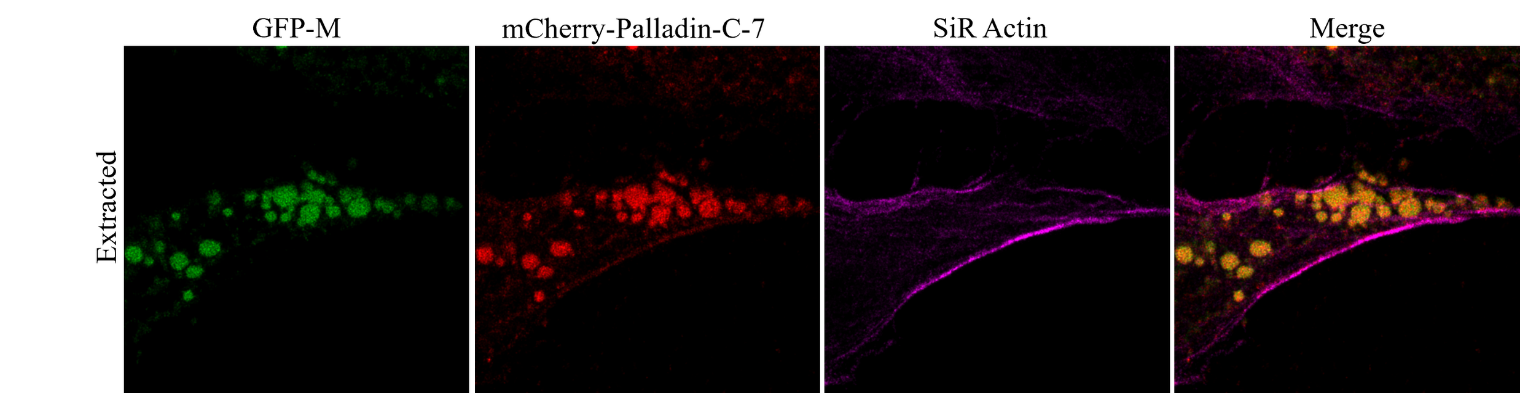


**Fig S2. Palladin associates and colocalizes with M and microfilaments in A549 cells.** Additional image for Fig 2. Cells were cultured overnight and transfected to express mCherry-Palladin-C-7 and GFP-M. Cytoskeleton enrichment was undertaken to determine the interaction between palladin and RSV M, and their interaction with the microfilament network. Transfected cells were either left untreated (images labelled unextracted) or treated (images labelled extracted) with enrichment buffers to remove soluble nuclear and cytosolic proteins. Cells were probed for visualization of the microfilament network (SiR Actin; in magenta). The colocalization of palladin (in red) and M (in green) is indicated in yellow (image labelled merge). Images were visualized by STED microscopy. Zoomed in image of extracted cells shown.





**Fig S3. Palladin forms complexes with M in infected and transfected cells.** Full western blot images for Fig 3. **(A)** Overnight sub-confluent monolayers were transfected to express mCherry-Palladin-C-7 and GFP-M proteins. Lysates were collected 24 h p.t.. Cells co-transfected to express mCherry-Palladin-C-7 and GFP-M were collected and sorted using FACS. Samples were used in a co-immunoprecipitation study to determine the palladin-M interaction through complex formation. Primary anti-mCherry antibody was either not added (labelled -) or added (2 µl) to lysates and immune complexes collected with EZview™ Red Protein G Affinity Gel. Unbound (labelled UB) and bound (labelled B) proteins were analyzed by western blot analysis and probed for GFP. **(B)** Overnight sub-confluent monolayers were transfected and collected as in **(A)**. Samples were analyzed by western blot before immunoprecipitation, and proteins of interest (labelled mCherry, mCherry-Palladin-C-7, GFP-M, and GFP) were detected by probing for mCherry **(i)** and GFP **(ii)**. The total size of the construct is calculated to be ~100 kDa; a band at this size was observed in all transfection experiments with this construct. **(C)** Overnight sub-confluent monolayers were infected with RSV A2 at a MOI of 1. Lysates were collected 16-18 p.i.. Cell lysates were used in a co-immunoprecipitation as in **(A)**. Primary anti-M antibody was either not added (labelled -) or added in increasing amounts (0.5 µl, 1 µl, and 2 µl) to lysates. Immune complexes collected in unbound and bound fractions were analyzed by western blot analysis and probed for palladin.


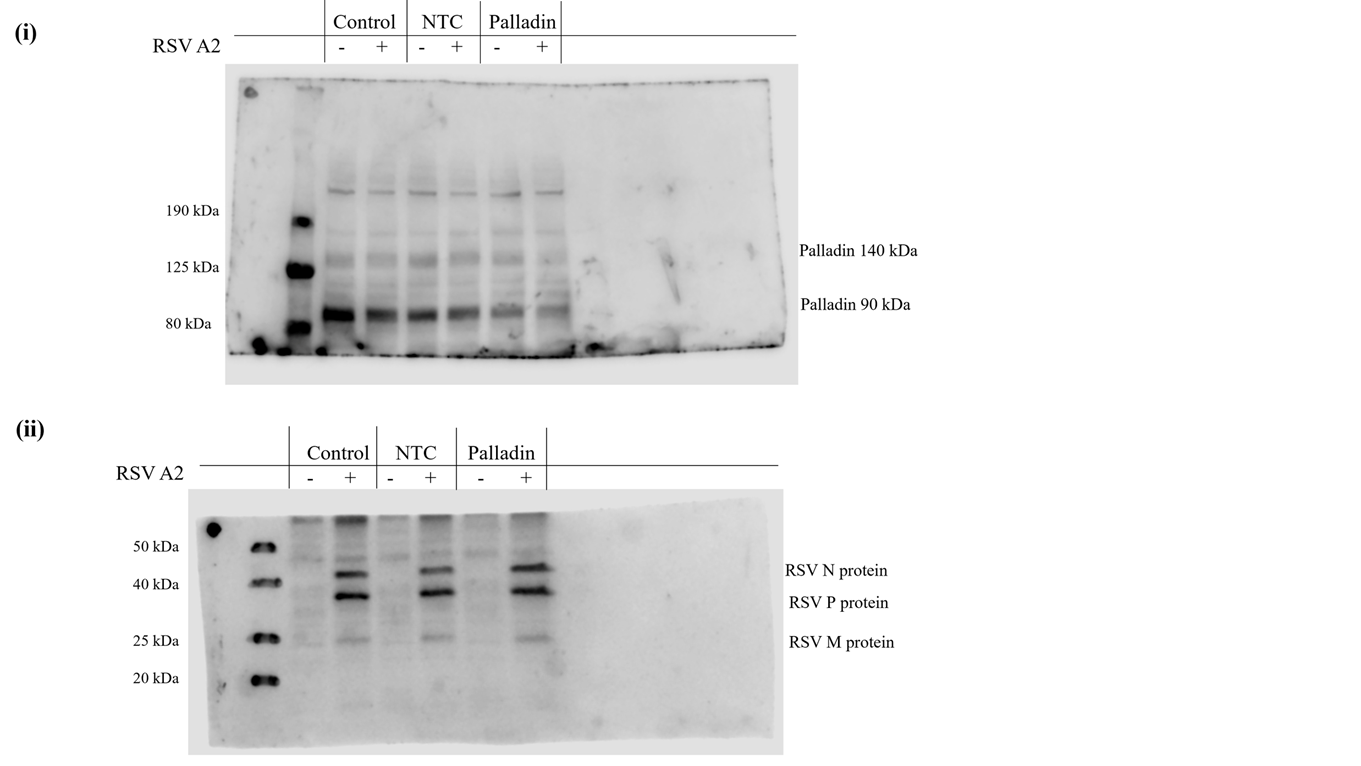
**Fig S4. Palladin is involved in RSV infection.** A549 cells were mock transfected (labelled No Treatment) or reverse transfected with Palladin siRNA or a Non-Targeting Control (labelled NTC) for 24 h. Cells were infected with RSV at an MOI of 1 before lysates were collected and analyzed by Western blot. Membranes were probed for the detection of palladin (panels labelled 140 kDa and 90 kDa) and RSV proteins (panel labelled RSV N, RSV P, and RSV M).


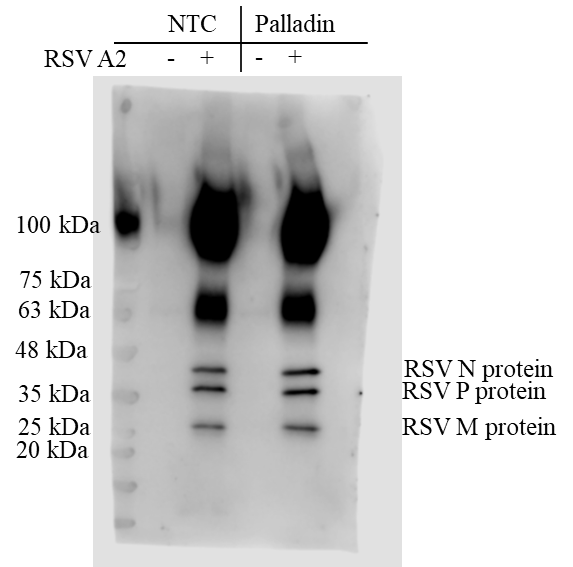


**Fig S5. Palladin is involved in RSV infection.** A549 cells were mock transfected (labelled No Treatment) or reverse transfected with Palladin siRNA or a Non-Targeting Control (labelled NTC) for 24 h. Cells were either left uninfected or infected with RSV at an MOI of 1 and supernatants or lysates were collected at 48 h p.i. Supernatants were collected and used in a sucrose cushion virus budding assay and purified virus was analzyed by western blot analysis. Membranes were probed for the detection of RSV proteins (N, P, and M).
